# *eif4ebp3l*—A New Affector of Zebrafish Angiogenesis and Heart Regeneration?

**DOI:** 10.3390/ijms231710075

**Published:** 2022-09-03

**Authors:** Lisa I. Born, Theresa Andree, Svenja Frank, Judith Hübner, Sandra Link, Marion Langheine, Anne Charlet, Jennifer S. Esser, Ralph Brehm, Martin Moser

**Affiliations:** 1 Department of Cardiology and Angiology, University Heart Center Freiburg, Faculty of Medicine, University of Freiburg, 79106 Freiburg, Germany; 2 Institute of Anatomy, University of Veterinary Medicine of Hannover, Foundation, 30173 Hannover, Germany

**Keywords:** myocardial infarction, zebrafish, heart, regeneration, 4E-BPs

## Abstract

The eukaryotic initiation factor 4E binding protein (4E-BP) family is involved in translational control of cell proliferation and pro-angiogenic factors. The zebrafish eukaryotic initiation factor 4E binding protein 3 like (*eif4ebp3l*) is a member of the 4E-BPs and responsible for activity-dependent myofibrillogenesis, but whether it affects cardiomyocyte (CM) proliferation or heart regeneration is unclear. We examined *eif4ebp3l* during zebrafish vascular development and heart regeneration post cryoinjury in adult zebrafish. Using morpholino injections we induced silencing of *eif4ebp3l* in zebrafish embryos, which led to increased angiogenesis at 94 h post fertilization (hpf). For investigation of *eif4ebp3l* in cardiac regeneration, zebrafish hearts were subjected to cryoinjury. Regenerating hearts were analyzed at different time points post-cryoinjury for expression of *eif4ebp3l* by in situ hybridization and showed strongly decreased *eif4ebp3l* expression in the injured area. We established a transgenic zebrafish strain, which overexpressed *eif4ebp3l* under the control of a heat-shock dependent promotor. Overexpression of *eif4ebp3l* during zebrafish heart regeneration caused only macroscopically a reduced amount of fibrin at the site of injury. Overall, these findings demonstrate that silencing of *eif4ebp3l* has pro-angiogenic properties in zebrafish vascular development and when *eif4ebp3l* is overexpressed, fibrin deposition tends to be altered in zebrafish cardiac regeneration after cryoinjury.

## 1. Introduction

Myocardial infarction (MI) is a form of ischemic heart disease (IHD) and thus one of the leading causes of death worldwide [1]. MI occurs mostly by occlusion of coronary vessels and results in ischemic necrosis of cardiomyocytes (CMs). The regenerative capacity of mature human CMs after necrosis is very limited [2,3]. Consequently, the damaged myocardium is mostly replaced by non-contractile fibrotic scar tissue, which stabilizes the heart ventricle, but leads to a loss of function and often to ischemic heart failure [4,5]. Current therapeutic options for heart failure do not include cardiomyocyte regeneration. They are limited to the alleviation of symptoms and delaying the progression of the disease whereas the ultimate goal would be the induction of endogenous cardiac tissue regeneration with replacement of the damaged myocardium. 

In contrast to adult humans and other mammalian species, adult zebrafish (*Danio rerio*) have a remarkable capability of complete cardiac regeneration after injury without formation of a persisting scar [6,7,8]. Although zebrafish phylogenetically belong to a different class than humans, they share about 70% of protein-encoding genes, similar developmental pathways and have a common evolutionary origin of heart tissue [9,10,11]. Based on this proximity to humans and its remarkable ability of heart regeneration, the zebrafish is an important animal model for studies of cardiovascular diseases. By establishing a zebrafish cardiac cryoinjury model in 2011 [7,8,12,13] a representative method has been found to mimic the ischemia-induced damage of cardiac tissue of human MI closely. Freezing 20–25% of the zebrafish heart ventricle with a pre-cooled cryoprobe results in a localized cell-death of all cardiac cell types, followed by tissue remodeling with transient fibrotic scar formation [7,8,12,13,14]. The first two phases of zebrafish heart regeneration post-injury, an initial inflammatory phase and a following reparative phase with scar formation are comparable to the human reparative program [15]. In zebrafish the determining difference is the replacement of the fibrotic scar by new functionally intact myocardium in the final regenerative phase [7,8,12,16]. In contrast, in humans the scar only maturates, but without being resolved [15]. During zebrafish heart regeneration, cardiac cells recapitulate developmental processes [17,18,19] including de-dedifferentiation and cardiomyocyte proliferation [17,20,21,22,23]. Re-expression of embryonic genes of heart and vascular development in mature CMs as well as in newly formed vessels is essential for complete heart regeneration [17,23,24,25,26].

The eukaryotic initiation factor 4E binding protein 3-like (*eif4ebp3l*) is the zebrafish homologue to the human eIF4EBP3, a member of the family of eukaryotic initiation factor 4E binding proteins (4E-BPs) [27,28]. 4E-BPs are key regulators of protein synthesis by binding to eIF4E during translation [29]. The binding to eIF4E inhibits formation of the eIF4F-complex and subsequently the cap-dependent translation initiation of various mRNAs [29,30]. Certain eIF4E-sensitive mRNAs encode for proteins that affect proliferation, e.g., vascular endothelial growth factor A (VEGFA) and fibroblast growth factor 2 (FGF2) [31,32,33,34]. In this context, eIF4E plays a crucial role in inducing angiogenesis and cell proliferation, which have been described mainly in tumorigenesis and carcinogenesis so far [31,32,33,34,35,36]. The 4E-BPs negatively affect the function of eIF4E and act as suppressors of proliferation and tumorigenesis [37,38,39]. Previous studies have focused on the role of 4E-BP1 and 4E-BP2, but little is known about 4E-BP3 and even less about the zebrafish homologue *eif4ebp3l*. Yogev et al. have shown that *eif4ebp3l* is the zebrafish 4E-BP with highest similarity (78%) to its human homologue and further plays a decisive role in activity–dependent myofibrillogenesis [40]. During muscle inactivity, *eif4ebp3l* is up-regulated and inhibits translation initiation of myocyte enhancer factor 2c (Mef2c), suppressing normal myofibrillogenesis and muscle fiber growth [40].

In this study we aimed to investigate the role of *eif4ebp3l* on angiogenesis during zebrafish vascular development and, additionally, its capability to affect zebrafish heart regeneration.

## 2. Results

### 2.1. Silencing of eif4ebp3l in Zebrafish Embryos Causes Vascular Sprouting

**Figure 1:**
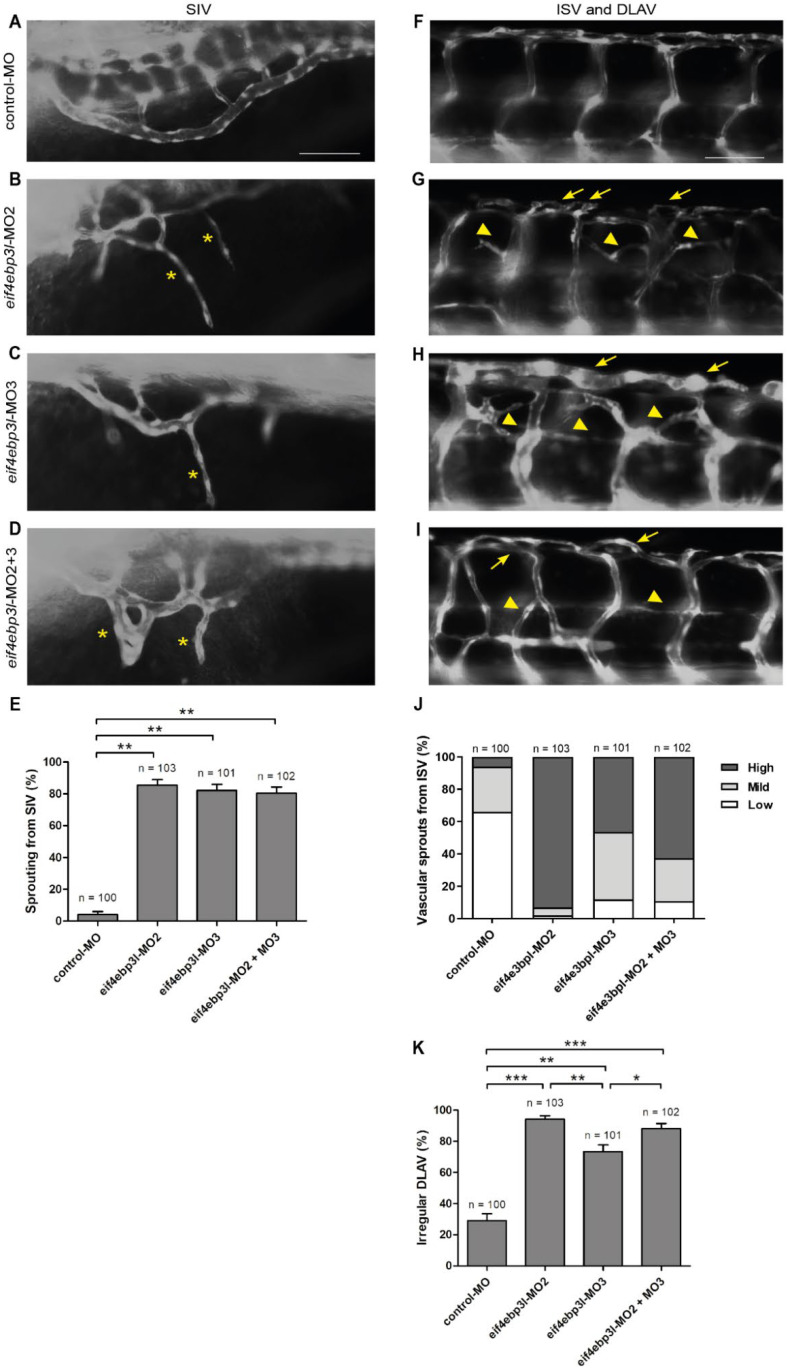
Loss of eukaryotic initiation factor 4E binding protein 3 like (*eif4ebp3l)* induced sprouting angiogenesis during zebrafish vascular development. (**A**–**I**) Lateral view of morpholino-injected *Tg(fli1:eGFP)* zebrafish at 94 h post fertilization (hpf). Anterior is to the left, abdominal (**A**–**D**) or trunk (**F**–**I**) regions are shown. Embryos were injected at 1–2 cell stage with control-MO (**A**), *eif4ebp3l*-morpholino 2 (MO2), *eif4ebp3l*-morpholino 3 (MO3) or a combination of the two. (**B**–**D**) In *eif4ebp3l* morphants the sub intestinal vein (SIV) was irregular and formed ventral additional vascular sprouts (yellow starlets). (**E**) Quantification of the formation of additional vascular sprouts from the SIV. (**F**–**I**) *Eif4ebp3l* morphants displayed vascular sprouting from the intersegmental vessels (ISVs) and within the dorsal longitudinal anastonomic vessel (DLAV). The additional sprouts formed cross links between neighboring ISVs (yellow arrowheads) and led to an increase of width in the DLAV (yellow arrows). (**J**) Quantification of the number of vascular sprouts of the ISV. Embryos were grouped in *low* phenotypes (one to two sprouts per ISV), *mild* (three to four sprouts per ISV) or *high* phenotypes (>five sprouts per ISV). (**K**) Quantification of *Tg(fli1:eGFP)* embryos with irregular DLAV. Comparison between the groups was made by using the Fisher‘s Exact test. *p*-value adjustments for multiple testing were performed by using the Bonferroni-Holm correction method. Values are mean ± SEM. * refers to *p* < 0.05, ** to *p* < 0.005 and *** to *p* < 0.0005. Scale bar = 50 µm.

In order to determine if *eif4ebp3l* modulates angiogenesis in zebrafish, as described for human members of the 4E-BP family, we used the endothelial-specific *Tg(fli:eGFP)* reporter fish for our morpholino experiments. *Eif4ebp3l* expression was specifically knocked down by injection of a splice blocking morpholino (MO2), a translation blocking morpholino (MO3) or the combination of the two in *Tg(fli:eGFP*) zebrafish embryos, respectively (Figure 1B–D,G–I). Injection of a standard–control morpholino (control-MO) served as a negative control (Figure 1A,F). Ectopic vascular sprouting from the sub-intestinal vein (SIV), the intersegmental vessels (ISV) and the dorsal longitudinal anastomotic vessel (DLAV) was observed in the *eif4ebp3l* MO groups, but not in the control (Figure 1). In time course experiments the strongest vascular phenotype was observed at 94 h post fertilization (hpf), thus we chose this time point for quantification. The SIV of *eif4ebp3l* MO injected embryos showed a ~20-fold increase of vascular sprouts compared to the control (Figure 1E). The sprouts from the SIV had a ventral orientation directed towards the yolk (Figure 1B–D). Their number varied between one and three per SIV. This vascular phenotype was nearly identical in the MO2 and MO3 groups (Figure 1E). Interestingly, embryos with injection of both *eif4ebp3l* morpholinos formed additional SIV loop structures between two neighboring vascular sprouts (Figure 1D). Furthermore, it was striking that in the double *eif4ebp3l* MO injected embryos the SIV itself was formed irregularly because of its ectopic sprouts. These pro-angiogenic effects of *eif4ebp3l* silencing were not limited to SIV but were also evident in the area of ISV. In control zebrafish embryos the ISVs were formed straight and regular between the somites with dorsal connection to the DLAV (Figure 1F). At the same stage, the ISV of *eif4ebp3l* morphants displayed ectopic vascular sprouting with formation of cross-links between neighboring ISVs (Figure 1G–I). The additional vessels were originated from the bottom or top of the ISV and varied in their number. According to the number of the new vascular sprouts, we grouped the embryos in *low*, *medium*, or *high* vascular phenotypes (Figure 1J). As a confirmation of the pro–angiogenic effect of *eif4ebp3l* silencing, *eif4ebp3l* morphant embryos showed a significantly higher prevalence for the *high* vascular phenotype compared to the control. However, *mild* and *low* phenotypes were also seen in the control embryos. Interestingly, blocking the splicing of *eif4ebp3l* by MO2 caused the highest number of new vascular sprouts from the ISV (Figure 1G,J). In addition to the changes in the SIV and ISV, the loss of *eif4ebp3l* also impaired the morphology of the DLAV. We observed significantly more irregular blood vessel formation of the DLAV in all three *eif4ebp3l* morpholino groups compared to control (Figure 1G–I,K). Additional sprouts led to an increase of the width and as a consequence to an irregular formation of DLAV (Figure 1G–I). The splice blocking morpholino MO2 particularly caused significantly more (*p* < 0.005) irregular vessel formation within the DLAV compared to all other groups (Figure 1K). Taken together, these morpholino data show that the silencing of *eif4ebp3l* has striking pro-angiogenic effects during zebrafish embryonic vascular development, suggesting that *eif4ebp3l* has anti-angiogenic properties.

### 2.2. eif4ebp3l Is Downregulated during Heart Regeneration

As our morpholino data of *eif4ebp3l* showed pro-angiogenic effects, we aimed to analyze *eif4ebp3l* expression during zebrafish heart regeneration. First, we performed a gene expression pattern screening of enhanced green fluorescent protein (eGFP)-positive, endothelial cells from *Tg(fli1:eGFP)* zebrafish hearts at three days post cryoinjury (dpci) or sham-OP (Figure S1). We aimed to investigate the expression pattern of *eif4ebp3l* during heart regeneration after cryoinjury and if *eif4ebp3l* expression is associated to endothelial cells. 

**Figure 2:**
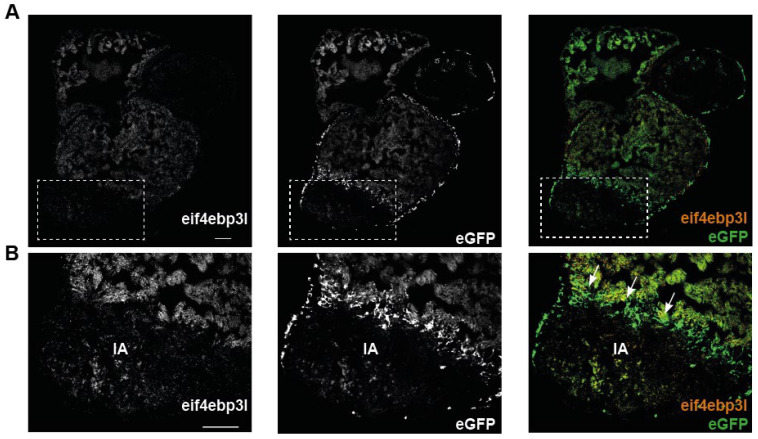
At day three after cryoinjury *eif4ebp3l* is downregulated in the injured area (IA). (**A**,**B**) Images of RNAscope in situ hybridizations (ISH) for *eif4ebp3l* (left panels, orange in the right panel) and eGFP (middle panels, green in the left panel) on cryosections of *Tg(fli1:eGFP)* zebrafish heart three days post cryoinjury (dpci). (**A**) Overview of the whole zebrafish heart; the IA is outlined with the dotted box. *Eif4ebp3l* was highly expressed in the healthy myocardium and downregulated in the IA. (**B**) Higher magnifications of the IA. eGFP was highly expressed in the border zone, where the formation of new endothelial vessels originated. *Eif4ebp3l* was co-located to these eGFP-positive endothelial cells in the border zone (white arrows), whereas in the center of the injured area its mRNA expression was strongly downregulated. Scale bar = 100 µm.

**Figure 3:**
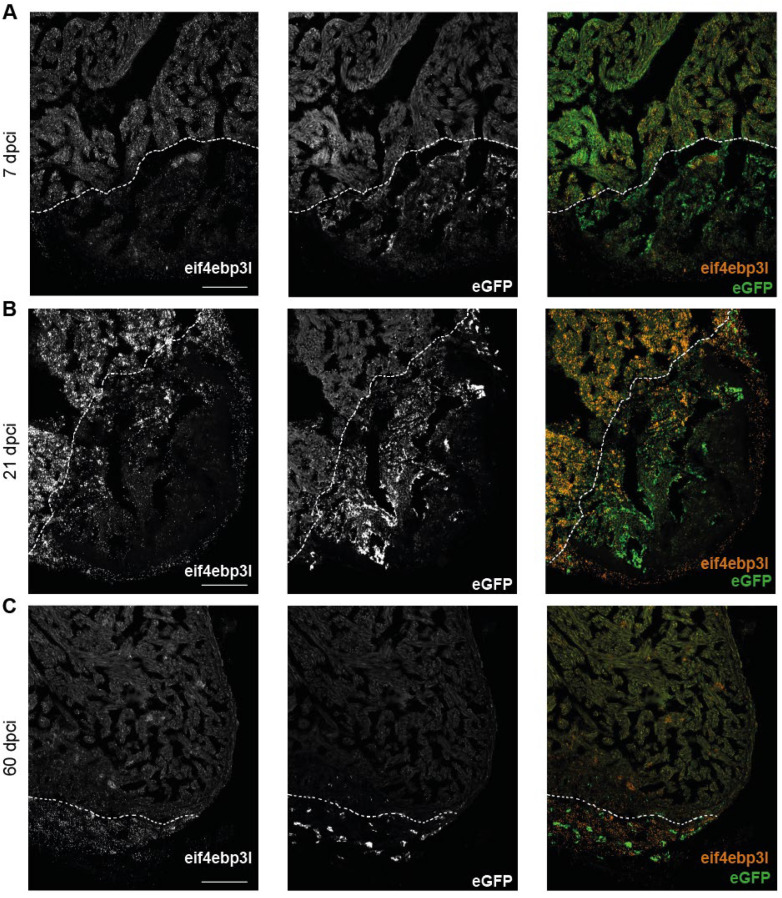
In the injured area the mRNA expression of *eif4ebp3l* decreased in the early phase of zebrafish heart regeneration. (**A**–**C**) RNAscope of the apex of *Tg(fli1:eGFP*) zebrafish heart at 7 dpci, 21 dpci and 60 dpci. In the merged image mRNA expression of eGFP is visualized in green and *eif4ebp3l* in orange. Dashed lines highlight the approximate border to the IA. (**A**) *Eif4ebp3l* was highly expressed in the healthy myocardium and downregulated in IA. (**B**,**C**) With progressive regeneration of the zebrafish heart, the expression of *eif4ebp3l* recovered in the IA and reached the mRNA expression level of the healthy myocardium at 60 dpci. Scale bar = 100 µm.

The analysis of the screen showed that *eif4ebp3l* was strongly downregulated in eGFP-positive endothelial cells of the injured area (Figure S1). To corroborate and expand the data from the screen, the spatial and temporal expression of *eif4ebp3l* during the process of zebrafish heart regeneration was investigated. Therefore, the RNAScope in situ hybridization technology was used on cryosections from *Tg(fli1:eGFP)* zebrafish hearts at 3 dpci, 7 dpci, 21 dpci and 60 dpci to visualize *eif4ebp3l* mRNA expression. To assess whether there is a co-localisation of *eif4ebp3l* to endothelial cells, eGFP was also detected using a specific RNAscope probe. Consistent with the data from the screen, *eif4ebp3l* was strongly downregulated in the injured area (IA) compared to the healthy myocardium at 3 dpci (Figure 2A,B). In addition, it showed spatial proximity to eGFP-positive, endothelial cells (Figure 2B, white arrows in the right panel). At all points in the time series, we observed that *eif4ebp3l* was much more strongly expressed in healthy myocardium than in the infarcted tissue during heart regeneration (Figure 3A–C). As the regeneration of the heart progressed, *eif4ebp3l* expression increased in the injured area and showed high expression levels in the border zone close to newly formed vessels (Figure 3A,B). At 60 dpci it nearly approached the expression level of the healthy myocardium (Figure 3C). In summary, *eif4ebp3l* showed spatial proximity to endothelial cells in the border zone of the injured area and revealed a specific expression pattern with downregulation in the early phase of injury and subsequent recovery during zebrafish heart regeneration.

### 2.3. Generation of Tg(fli1:eGFP/hsp70l:eif4ebp3l-p2A-tdTomato) for Heat-Shock Inducible eif4ebp3l Overexpression

**Figure 4:**
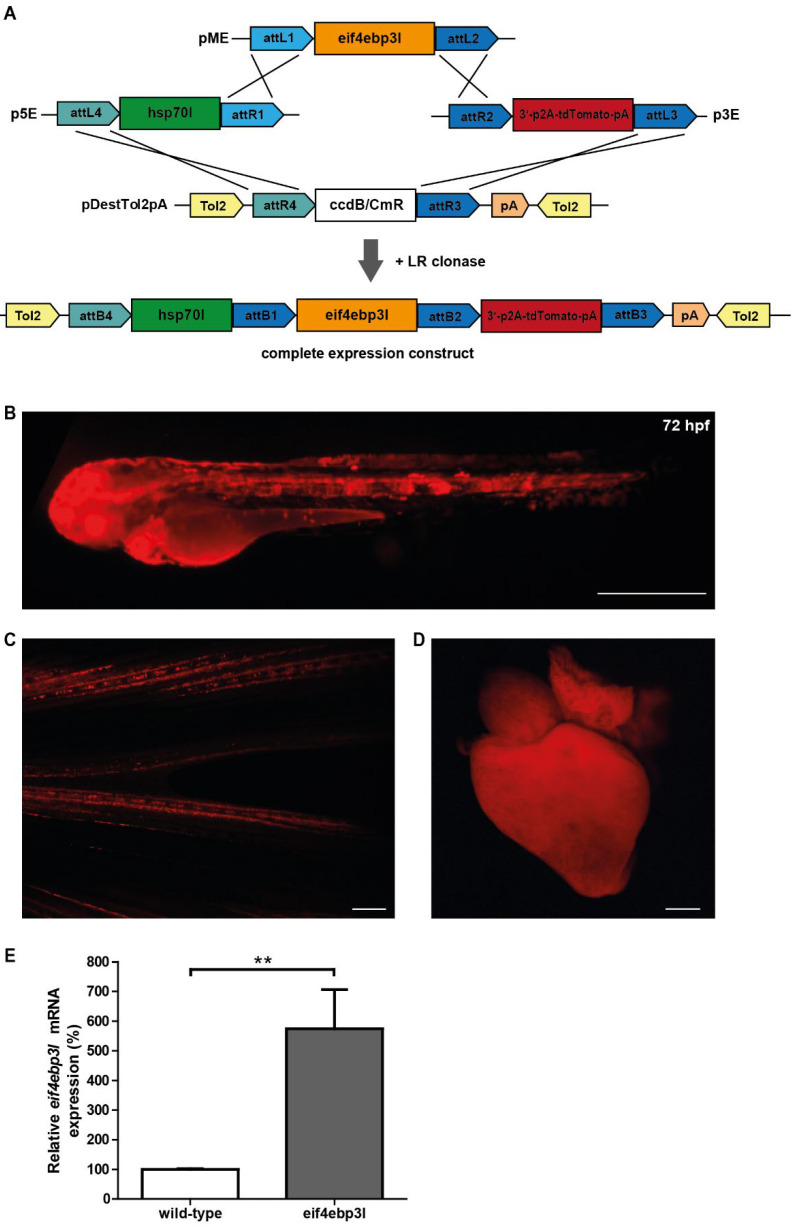
Heat-shock inducible overexpression of *eif4ebp3l.* (**A**) Schematic diagram of *eif4ebp3l* transgene and its cloning strategy used to achieve the heat-shock inducible overexpression of *eif4ebp3l*. (**B**) Embryo of the transgenic zebrafish strain *Tg(fli1:eGFP/hsp70l:eif4ebp3l-p2A-tdTomato)* 42 h after heat-shock. (**C**,**D**) Caudal fin and heart of an adult *Tg(fli1:eGFP/hsp70l:eif4ebp3l-p2A-tdTomato*) zebrafish 44 h and 24 h after heat-shock treatment. (**E**) Quantification of *eif4ebp3l* mRNA expression of *Tg(fli1:eGFP/hsp70l:eif4ebp3l-p2A-tdTomato*) zebrafish embryos and their wild-type siblings after heat-shock treatment by qRT-PCR. Embryos were heat-shocked at 30 hpf. Values are mean ± SEM and ** refers to *p* < 0.005. *n* = 300 per group. Scale bar = 500 µm.

To obtain functional data regarding *eif4ebp3l*, we generated a new transgenic zebrafish strain *Tg(fli1:eGFP/hsp70l:eif4ebp3l-p2A-tdTomato).* In this strain, global overexpression of *eif4ebp3l* is driven by the heat-shock promoter *hsp70l* and linked to the red fluorescent reporter protein *tdTomato* (Figure 4A–D). After heat-shock treatment, *eif4ebp3l* overexpressing zebrafish displayed a red fluorescence (Figure 4B–D). For quantification of *eif4ebp3l* mRNA levels, we used quantitative Real-Time-PCR (qRT-PCR). Upon heat-shock, *Tg(fli1:eGFP/hsp70l:eif4ebp3l-p2A-tdTomato)* embryos expressed a five-fold higher level of *eif4ebp3l* mRNA than their wild-type siblings (Figure 4E). Together, these data show that we successfully established a new transgenic zebrafish strain with heat-shock inducible overexpression of *eif4ebp3l*.

### 2.4. Effects of eif4ebp3l Overexpression on Scar Formation and Composition after Cryoinjury

**Figure 5:**
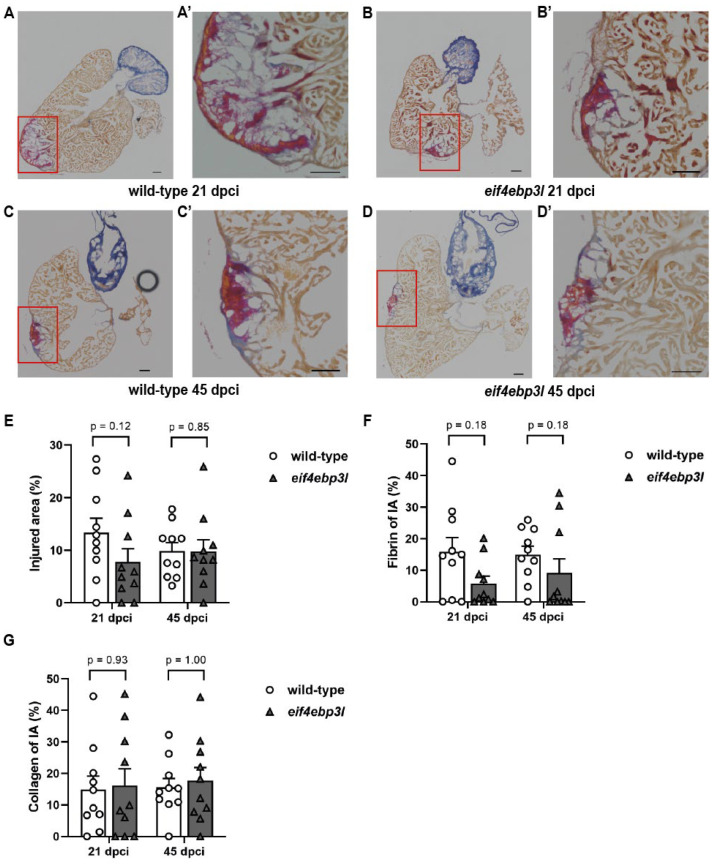
Effects of *eif4ebp3l* overexpression on scar formation after cryoinjury. (**A**–**D’**) Acid fuchsin orange G (AFOG) staining of wild-type *Tg(fli1:eGFP)* and *Tg(fli1:eGFP/hsp70l:eif4ebp3l-p2A-tdTomato)* zebrafish hearts at 21 dpci and 45 dpci. Intact myocardium is stained in orange, fibrin in red and collagen in blue. The injured area (IA) is marked by the red box. (**A’**–**D’**) Magnified view of the IA. (**E**–**G**) Quantification of the IA and its composition. Values are mean ± SEM, *n* = 10 per group and day post cryoinjury. Scale bar = 100 µm.

To determine the impact of heat-shock induced *eif4ebp3l* overexpression on zebrafish heart regeneration, we compared the size and composition of the injured area between *Tg(fli1:eGFP/hsp70l:eif4ebp3l-p2A-tdTomato*) zebrafish and their wild-type (WT) siblings after cryoinjury (Figure 5A–G). *Tg(fli1:eGFP*) zebrafish from the same clutch, but without the *eif4ebp3l* transgene, were considered as WTs and treated with the same heat-shock protocol. Therefore, the time points 21 dpci and 45 dpci in the reparative phase of regeneration were chosen. According to the size, WTs and *Tg(fli1:eGFP/hsp70l:eif4ebp3l-p2A-tdTomato)* fish revealed similar values at 21 dpci (Figure 5A–B’,E). Intriguingly, the amount of fibrin was macroscopically clearly decreased in *Tg(fli1:eGFP/hsp70l:eif4ebp3l-p2A-tdTomato)* hearts at 21 dpci (Figure 5B,B’,F), but statistical significance could not be shown due to the high dispersion of the values collected. The proportion of collagen was approximately equal in both groups at 21 dpci (Figure 5G). At 45 dpci we observed no striking differences regarding the size of the IA and the proportion of collagen between *Tg(fli1:eGFP/hsp70l:eif4ebp3l-p2A-tdTomato)* hearts and WT hearts (Figure 5C–D’,E,G). However, *Tg(fli1:eGFP/hsp70l:eif4ebp3l-p2A-tdTomato)* fish again displayed a macroscopically lower proportion of fibrin at 45 dpci compared to their WT siblings (Figure 5F).

In conclusion, zebrafish overexpressing *eif4ebp3l* showed no significant differences in the late cardiac regeneration compared to WTs, but tend to have macroscopically lower levels of fibrin in the injured area.

### 2.5. Cardiomyocyte Proliferation after Cryoinjury

**Figure 6:**
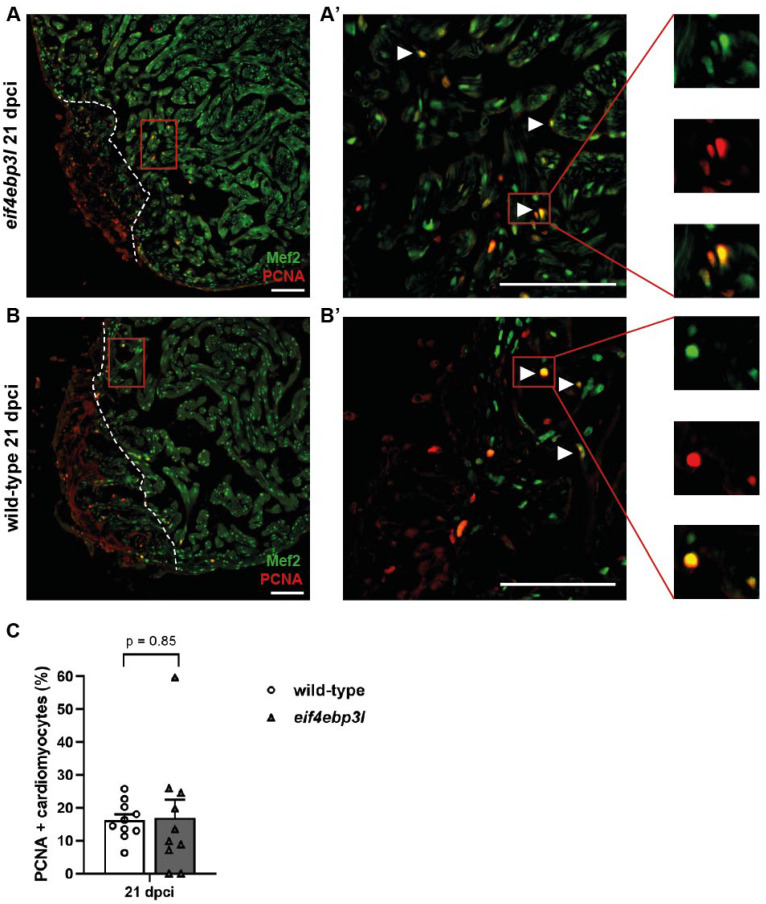
Comparison of cardiomyocyte (CM) proliferation of *Tg(fli1:eGFP/hsp70l:eif4ebp3l-p2A-tdTomato)* and wild-type siblings at 21 dpci. (**A**–**B’**) Representative immunofluorescence images of cardiac tissue of wild-type and *Tg(fli1:eGFP/hsp70l:eif4ebp3l-p2A-tdTomato*) at 21 dpci. Mef2 marks CM nuclei in green and PCNA nuclei of proliferating cells in red. PCNA–positive CMs are indicated with white arrowheads, and insets are magnified views of the marked cells. Dashed lines highlight the approximate border of the injured area. (**C**) Quantification of proliferating CMs expressing PCNA within 150 µm from the injury border. Values are mean ± SEM, *n* = 10 per group. Scale bar = 100 µm.

To assess if cardiomyocyte proliferation is controlled by *eif4ebp3l* overexpression during zebrafish heart regeneration, we performed double immunostainings with anti-Mef2, a specific CM marker, and anti-proliferating cell nuclear antigen (PCNA), a marker of proliferating cells in heart sections of *Tg(fli1:eGFP/hsp70l:eif4ebp3l-p2A-tdTomato)* and WT zebrafish at 21 dpci (Figure 6A,B). CMs expressing Mef2 and PCNA (Figure 6A’,B’, white arrowheads and insets) were considered as proliferating CMs and mainly detected in the border zone of the injured area. The number of proliferating CMs showed a similar level (~15% of all Mef2+ cells in the border area) in WT and *Tg(fli1:eGFP/hsp70l:eif4ebp3l-p2A-tdTomato)* fish (Figure 6C). 

## 3. Discussion

Our study revealed three new insights into the translational control of angiogenesis and cell proliferation by *eif4ebp3l* during zebrafish vascular development and heart regeneration: first, silencing of *eif4ebp3l* had strong pro-angiogenic effects during zebrafish embryonic angiogenesis; second, it had a specific gene expression pattern during heart regeneration depending on the phase of heart regeneration, and third, *eif4ebp3l* overexpression tended to affect fibrin deposition on the late cardiac regeneration. 

During zebrafish vascular development, sprouting angiogenesis is an essential process regarding the formation of the secondary blood vessels such as the ISVs and the DLAV [41]. Various growth factors, e.g., VEGFA, trigger this process and promote angiogenesis [42]. Here, we demonstrated that *eif4ebp3l* may also play a role in the complex process of angiogenesis. Injections of splice and translation blocking MO both significantly caused formation of new vascular sprouts in zebrafish embryos at 94 hpf. We assessed these effects as *eif4ebp3l*-specific, because they were more frequently observed in all three groups with injections of *eif4ebp3l* morpholinos compared to the control. Further, off-target effects were minimized by co-injecting a p53 morpholino [43]. Consequently, silencing of *eif4ebp3l* led to additional vascular sprouts within the SIV, ISVs and the DLAV and, thus, has specific pro-angiogenic effects. These findings show that *eif43bp3l* modulates angiogenesis here in the novel context of zebrafish embryonic vascular development. So far, this has only been described for its human homologue 4E-BP3 in the context of angiogenesis and cell proliferation during tumorigenesis [37,38,39].

Since re-expression of embryonic genes of heart and vascular development plays a crucial role in zebrafish heart regeneration [17,18,21,23,24,25,26], we analyzed the expression pattern of *eif4ebp3l* during cardiac regeneration after cryoinjury. An initial screen (Figure S1) showed strongly decreased levels of *eif4ebp3l* expression in eGFP-positive cells of the injured area compared to the healthy myocardium at 3 dpci. We confirmed this finding by in situ hybridization and demonstrated close co-expression of *eif4ebp3l* with eGFP-positive endothelial cells in the border zone of the injured area. Thus, we hypothesize that our findings from the morpholino data might be transferred to the process of zebrafish heart regeneration. We further looked at changes of the expression pattern of *eif4ebp3l* with progressive heart regeneration and had two major findings: First, in the early phase of zebrafish heart regeneration (3 dpci until 21 dpci), *eif4ebp3l* is strongly downregulated in the injured area. In this phase an early and fast revascularization of the injured area is essential to build a vascular scaffold and to promote CM proliferation and migration along this scaffold for the re-population of the wound [24,44]. Since angiogenesis and proliferative processes peak in this period [7,8,12], downregulation of *eif4ebp3l* in the injured area seems to be beneficial to cardiac regeneration. This is consistent with our observations from the morpholino experiments, in which silencing of *eif4ebp3l* had pro-angiogenic effects and promoted sprouting angiogenesis. Second, at 21 dpci mRNA expression of *eif4ebp3l* increased in the injured area and reached baseline level of the healthy myocardium at 60 dpci. From day 21 after cryoinjury, the injured area is completely covered by vasculature [8,44] and most proliferative processes such as CM proliferation or proliferation of the endocardium are nearing their end [45,46]. For balancing cell proliferation and prevention of unrestrained, cancerous cell growth after injury, the up-regulation or general expression of anti-proliferative genes such as *runx1*, is detected [47,48]. Because of its anti-proliferative effects, increased expression of *eif4ebp3l*, beginning at 21 dpci, might also take part in controlling the proliferative injury response in zebrafish heart regeneration.

We hypothesized that overexpression of *eif4ebp3l* could lead to a slower and incomplete cardiac regeneration, because it suppresses translation of proliferative proteins such as FGF or VEGFA [27,28,31,32,33,34]. In order to test this hypothesis, we successfully established a new transgenic zebrafish strain with a heat-shock promotor driven global overexpression of *eif4ebp3l*. After injury, remodeling of the extracellular matrix (ECM) and fibrin deposition are essentially required for stimulating CM proliferation and regeneration in the zebrafish heart [49]. In our study, we observed that *Tg(fli1:eGFP/hsp70l:eif4ebp3l-p2A-tdTomato)* fish tend to have a lower proportion of fibrin in the injured area compared to wild-types at both time points. However, at day 21 after injury, we found similar amounts of PCNA positive CMs in *eif4ebp3l* overexpressing hearts and wild-type hearts. In agreement with previous studies, the average number of proliferating CMs was small, as CM proliferation peaks at seven dpci and approaches pre-injury levels at around 30 dpci [7,8,12,46]. Further studies are needed to evaluate if these observations are only limited to late cardiac regeneration or if *eif4ebp3l* affects heart regeneration at an earlier time point. Furthermore, it should be considered that overexpression of *eif4ebp3l* might not be sufficient for a significant effect, as it is only one of many players of the complex system of translational control by the 4E-BPs and eIF4E. Besides other functions, *eif4ebp3l* also competes with eIF4G for the binding site of eIF4E [50,51] and its activity is regulated via phosphorylation by the mTOR complex 1 (mTORC1) [52,53]. For example, Yogev et al. showed that overexpression of *eif4ebp3l* only had an effect on myofibrillogenesis in the presence of muscle inactivity, when mTORC1 activity is reduced and hyperphosphorylation of *eif4ebp3l* is prevented [40]. Altogether, this might explain the mild effects of *eif4ebp3l* overexpression on the late phase of zebrafish cardiac regeneration. 

In conclusion, our study shows a beneficial response to silencing of *eif4ebp3l* in angiogenesis during zebrafish development and, consistent with this, we detected downregulation of *eif4ebp3l* in the early, proliferative phase of zebrafish heart regeneration. As an effect of *eif4ebp3l* overexpression, we only observed a tendency of a decreased proportion of fibrin in the injured area after cryoinjury in the late zebrafish cardiac regeneration.

## 4. Materials and Methods

### 4.1. Zebrafish Strains and Husbandry

Zebrafish wild–type TL strain and transgenic lines *Tg(fli1:eGFP)* and *Tg(fli1:eGFP/hsp70l:eif4ebp3l-p2A-tdTomato)* were raised and maintained in the zebrafish facility at the university heart center (UHZ) Freiburg. They were housed at 26 °C water temperature and a 14:10 h light dark cycle.

### 4.2. Embryo Raising

Zebrafish embryos were raised and staged as previously described [54,55]. For embryo treatment, fertilized eggs were kept in 0.3× Danieau’s medium with 0.003% 1-phenyl-2-thiourea (Sigma Aldrich, Steinheim, Germany) at 28 °C from 24 hpf in order to suppress pigmentation. 

### 4.3. Morpholino Injection

For morpholino experiments, one or two cell stage embryos were injected with 1 nL of the indicated amounts of MO diluted in ddH_2_O. All MOs were synthesized by Gene Tools, LCC, Philomath, USA. We used the following MO sequences:

*eif4ebp3l* splice blocking MO2: 5′-ATAGTGAGAGTGGGTCTTACCGCCA-3′ (0.25 mM) *eif4ebp3l* translation blocking MO3: 5′-TTGTGGACATCGTGCGTCAAAATGC-3′ (0.25 mM)
*p53* MO: 5′-GCGCCATTGCTTTGCAAGAATTG-3′ (0.375 mM)

The standard-control MO (0.25 mM) was used as a negative control. To avoid off-target effects the p53 morpholino was co-injected. After injection, all eggs were raised in 0.3× Danieau’s medium at 28 °C until further use.

### 4.4. Generation of Heat-Shock Inducible Tg(fli1:eGFP/hsp70l:eif4ebp3l-p2A-tdTomato)

At first, the open reading frame of *eif4ebp3l* was amplified using a proofreading polymerase and the cDNA extracted from 48 hpf old zebrafish as the template. The utilized primers are listed below:

Forward-Primer: 5′-ATGTCCACAAACACGCAGCAG-3′
Reverse-Primer: 5′-TCAGATGTCCATCTCAAACTGGCTG-3′

The gene sequence was then cloned into the *pCR2.1-TOPO*-Vector and amplified after transformation in bacteria. The purified vector was subsequently PCR-amplified using the following primers containing attB1/2 sites. The att sites are written in bold. 

Forward-Primer:

5′-**GGGGACAAGTTTGTACAAAAAAGCAGGCT**TCGCCACCATGTCCACAAACACGCAGCAG-3′

Reverse-Primer:

5′-**GGGGACCACTTTGTACAAGAAAGCTGGGT**AGATGTCCATCTCAAACTGGCTGTCG-3′

By using the Gateway System (Invitrogen, Carlsbad, Germany), the freshly synthesized PCR–product was combined with a pDONR221 vector in a BP reaction to generate the middle entry clone *pME-eif4ebp3l*. Afterwards, it was recombined with the *pDestTol2pA2* vector, the *p5E-hsp70l* and the *p3E-p2A-tdTomato* vector in a LR reaction to create the expression construct. The construct containing the gene sequence of *eif4ebp3l*, the tol2 sites, the heat–shock protein *hsp70l* and the reporter protein *tdTomato* was then injected into single-cell-staged *Tg(fli1:eGFP)* embryos. Simultaneously the mRNA of a transposase was injected, which allows the integration of the expression construct into the genome of the zebrafish. The transposase mRNA was generated by transcription of the pCS2FA-Transposase vector in vitro using the mMessage mMachine transcription Kit (Invitrogen, Carlsbad, Germany). The *tdTomato* protein served as an easy screening way for germline transgenic animals. The correctness of the *pCR2.1-TOPO-eif4ebp3l* vector and the pME-*eif4ebp3l* vector were verified by sanger sequencing by Eurofins Genomics, Ebersberg, Germany.

### 4.5. Heat–Shock Treatment 

To induce overexpression of *eif4ebp3l,* zebrafish embryos and adult fish of the transgenic line *Tg(fli1:eGFP/hsp70l:eif4ebp3l-p2A-tdTomato)* and their wild-type siblings were heat–shocked. The heat–shock treatment of the embryos was performed in an incubator or water bath at the age of 30 hpf for 1 h at 38 °C. Adult zebrafish were heat–shocked for 1 h at 38 °C in a water bath. The treatment was done once before cryoinjury and then three times per week after cryoinjury until the hearts were collected.

### 4.6. Cryoinjury

Cryoinjury of *Tg(fli1:eGFP)* and *Tg(fli1:eGFP/hsp70l:eif4ebp3l-p2A-tdTomato)* fish was performed as previously described [56]. The hearts of *Tg(fli1:eGFP)* zebrafish were collected at the time points 3 dpci, 7 dpci, 21 dpci and 60 dpci; hearts of *Tg(fli1:eGFP/hsp70l:eif4ebp3l-p2A-tdTomato)* fish were collected at 21 dpci and 45 dpci. For cryosectioning, all hearts were fixed in 4% paraformaldehyde in PBS at 4 °C for 48 h, dehydrated in methanol, stored in 30% sucrose–PBS solution at 4 °C overnight and embedded in freezing medium TissueTek OCT Compound (Sakura, Alphen am Rhein, The Netherlands). All probes were sectioned with a Leica CM 1950 cryostat in 10 µm slides.

### 4.7. In Situ Hybridization 

The in situ hybridization was performed on cryosections from cryoinjured hearts of *Tg(fli1:eGFP*) zebrafish. Different types of in situ hybridizations were conducted: first the in situ hybridization with Digoxigenin–labeled RNA probes was undertaken according to Jostarndt et al. [57], with some modifications. Second, in situ hybridization with fluorophor–labeled RNA probes using the RNAScope ^®^ (ACD Biosciences, Newark, DE, USA) technology was undertaken as per the manufacturer’s instructions.

### 4.8. RNA Isolation and Quantitative Real-Time PCR (qRT-PCR)

For quantification of *eif4ebp3l* overexpression, RNA was extracted from *Tg(fli1:eGFP/hsp70l:eif4ebp3l-p2A-tdTomato)* zebrafish embryos and their wild-type siblings after heat–shock induction using TRIzol lysis reagent (Qiagen Sciences, Germantown, MD, USA) according to the manufacturer’s instruction. Reverse transcriptions were performed with an iScript cDNA-Kit (Bio-Rad, Feldkirchen, Germany) by applying 0.5 µg RNA following the manufacturer’s instructions. Quantitative RT-PCR analysis was done by using IQ SybrGreen 2x Supermix and the iCycler real-time PCR detection system (Bio-Rad, Feldkirchen, Germany). The following primer sequences were used:

*eif4ebp3l*_fwd: 5′-ATGTCCACAAACACGCAGCAG-3′
*eif4ebp3l*_rev: 5′-TCAGATGTCCATCTCAAACTGGCTG-3′
*efl1α*_fwd: 5′-CATCTGATCTACAAATGCGGTGG-3′
*efl1α*_rev: 5′-CTGGTCTCGAATTTCCAGAGAG-3′

The primers were purchased from Eurofins Genomics, Ebersberg, Germany. The housekeeping gene zebrafish elongation factor 1 alpha (efl1α) was used as an internal control. The qRT-PCR program was as follows: initial denaturation at 94 °C for 1 min followed by 50 cycles of denaturation at 94 °C for 30 s, annealing at 55 °C for 30 s and extension at 72 °C for 30 s. Fluorescence was measured after each extension step. A final elongation was performed at 72 °C for 5 min before a melting curve was acquired. Relative quantification of *eif4ebp3l* expression was calculated by the ∆∆CT method [58,59].

### 4.9. Histological Stainings

Different histological stainings were performed to determine the impact of *eif4ebp3l* overexpression during heart regeneration. The Acid Fuchsin Orange G (AFOG) staining was done as described [6]. The ventricle and infarct area were measured using Zen 3.1 software (blue edition, Carl Zeiss microscopy GmbH, Oberkochen, Germany). The percentage of infarct size relative to the ventricle as well as the percentage of fibrin and collagen were calculated.

For immunofluorescence, cryosections were immersed in boiling citric acid buffer (10 mM) for 30 min at 96 °C–99 °C for antigen retrieval. After short washes in PBT, the sections were blocked in 5% BSA, 5% NGS, 5% donkey serum, 0.5% Triton X-100, 20 mM MgCl_2_ in PBS (Blocking buffer) for 1 h at room temperature. The sections were incubated with the antibodies anti-PCNA PC10 (mouse; abcam, 1:10,000) and anti-Mef2 (rabbit; boster biological technology, 1:200) in sequence overnight at 4 °C. Following washes with PBS, the sections were incubated with the secondary antibodies Alexa 647 (goat; invitrogen, 1:200) and Alexa 555 (donkey; invitrogen, 1:200) for 2 h at room temperature. Finally, the slides were mounted with DAPI mounting medium (Carl Roth, Karlsruhe, Germany). For quantification Mef2+ and Mef2+/PCNA+ cells were counted manually in a range of 150 µm from the border area of the infarct. To determine the percentage of proliferating CMs (considered as Mef2+/PCNA+ cells), five regions of interest (ROIs) were averaged in the border area of each heart.

### 4.10. Image Acquisition and Processing

Zebrafish embryos were dechorionated, anesthetized with tricaine (4 mg/mL), and placed on a 3% agarose plate for imaging. The images were taken with the Zeiss Axiovert A1 microscope (Carl Zeiss microscopy, Oberkochen, Germany), equipped with a Zeiss AxioCam HR (Carl Zeiss microscopy, Oberkochen, Germany).

After collecting, the hearts were directly imaged with the camera Leica DFC 450C ((Leica microsystems, Wetzlar, Germany) of the binocular microscope Leica MZ10 F (Leica microsystems, Wetzlar, Germany) to determine the injured area of the heart after cryoinjury. Transmitted light and RNAScope ^®^ microscopy were performed with a Zeiss Axio Imager Z2 microscope (Carl Zeiss microscopy, Oberkochen, Germany), with Axiocam 305 color and Axiocam 506 mono cameras (Carl Zeiss microscopy, Oberkochen, Germany). Immunofluorescence images were acquired using a Leica DMi8 inverse microscope (Leica microsystems, Wetzlar, Germany) with a Leica K5 camera (Leica microsystems, Wetzlar, Germany) with LAS X 3.75 software.

### 4.11. Statistical Analysis and Quantification

Statistical analysis was performed using SAS-Enterprise Guide (Version 7.15, SAS Institute Inc., Cary, NC, USA) and GraphPad Prism 5.01 (GraphPad Software, LLC, San Diego, CA, USA). Data were tested for normal or non-normal distribution. For normally distributed data, a Student’s *t*-test for comparison of two groups was performed. If the data were not normally distributed, a Mann-Whitney *U* test was performed. Phenotypes of *eif4ebp3l* knockdown in zebrafish embryos were compared by using the Fisher’s Exact Test. The Bonferroni-Holm correction method was used to adjust *p*-values for multiple testing. Results were considered statistically significant at *p* < 0.05.

## Data Availability

The data presented in this study are available in this article.

## Supplementary Materials

The following supporting information can be downloaded at: https://www.mdpi.com/article/10.3390/ijms231710075/s1.
