# Supplementary material for: eif4ebp3l—A New Affector of Zebrafish Angiogenesis and Heart Regeneration?
Source: Int J Mol Sci. 2022 Sep 3;23(17):10075. doi: 10.3390/ijms231710075 (PMC9456460; doi:10.3390/ijms231710075)
Supplement: Supplementary file 1 [file ijms-23-10075-s001.zip › ijms-1865331-supplementary.pdf]

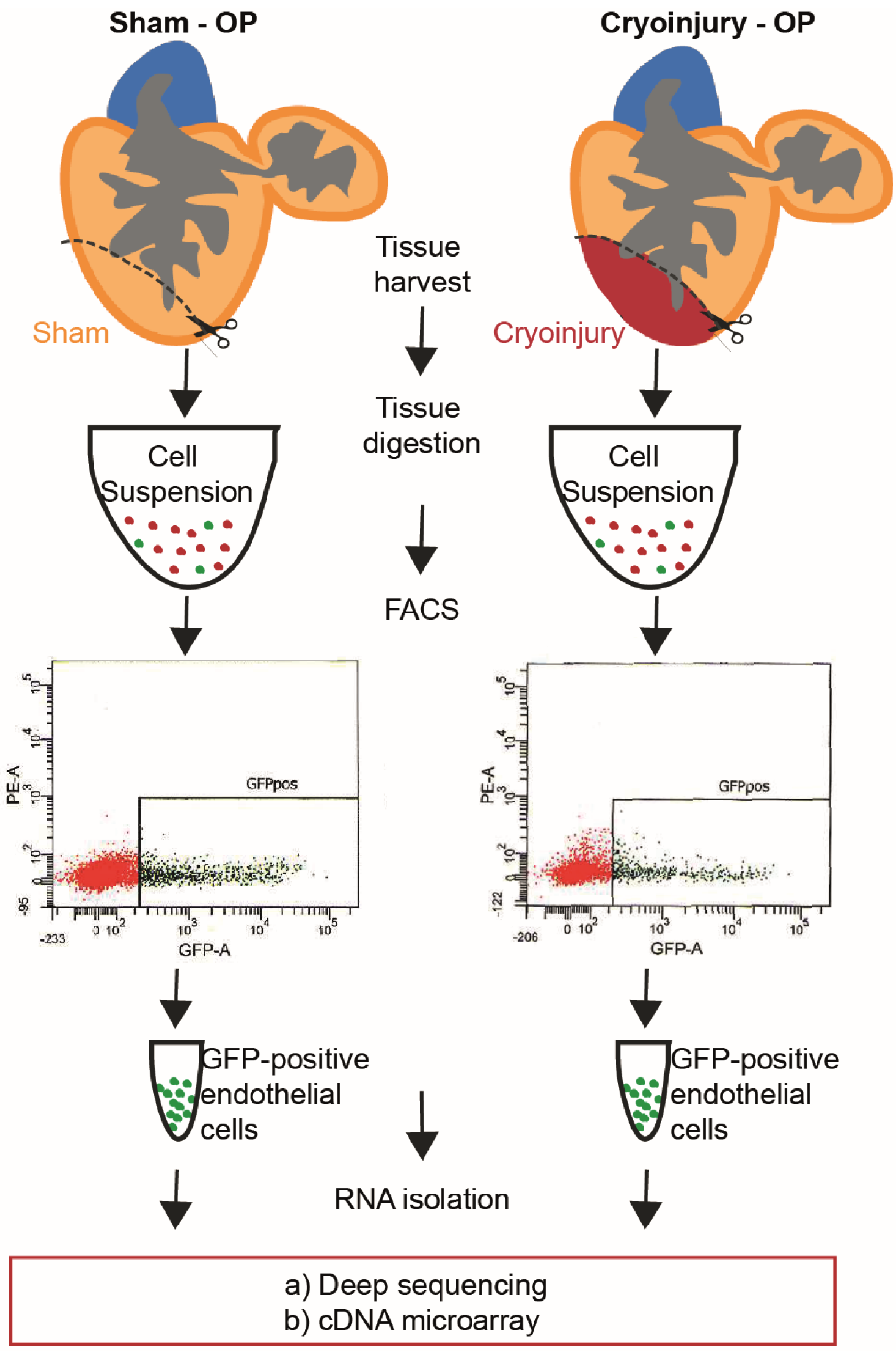

Figure S1. Analysis of GFP-positive endothelial cells identifies *eif4ebp3l* gene expression in the injured zebrafish heart. Schematic overview of gene expression screening procedure of cells from zebrafish heart at day 3 after cryoinjury or sham OP. Cells from the apex of *Tg(fli1:eGFP)* zebrafish heart were sorted by FACS for GFP-positivity and their gene expression patterns were analyzed by deep sequencing and cDNA microarray. *Eif4ebp3l* was detected as one of the gene candidates with strong downregulation in the in injured area.
